# Differential NMR Lipoprotein Profiles and Prediction of Insulin Resistance and Metabolic Syndrome by LP-IR Among Adult Chronic Hepatitis B Patients

**DOI:** 10.3390/jcm14207405

**Published:** 2025-10-20

**Authors:** Karen J. Campoverde Reyes, Javier Guevara, Hamidreza Karimi-Sari, Daniela Goyes, Kamolyut Lapumnuaypol, Pir A. Shah, Satinder P. Kaur, Margery A. Connelly, Z. Gordon Jiang, Daryl T.-Y. Lau

**Affiliations:** 1 Liver Center, Division of Gastroenterology and Hepatology, Department of Medicine, Beth Israel Deaconess Medical Center, Harvard Medical School, Boston, MA 02215, USA; kcampove@bidmc.harvard.edu (K.J.C.R.); jguevaraalvarez@bwh.harvard.edu (J.G.); daniela.goyes@yale.edu (D.G.); kamolyut.lapumnuaypol@sanfordhealth.org (K.L.); pirshah@creighton.edu (P.A.S.); satinder.kaur@airliquide.com (S.P.K.); zgjiang@bidmc.harvard.edu (Z.G.J.); 2 Metabolism and Neuroendocrine Unit, Massachusetts General Hospital, Harvard Medical School, Boston, MA 02114, USA; 3 School of Medicine, Johns Hopkins University, Baltimore, MD 21218, USA; hkarimi1@jhmi.edu; 4 Labcorp, Morrisville, NC 27560, USA; connem5@labcorp.com

**Keywords:** chronic hepatitis B, MASLD, nuclear magnetic resonance spectroscopy, lipoproteins, insulin resistance, LP-IR

## Abstract

**Background/Objectives**: Chronic hepatitis B and metabolic dysfunction-associated steatotic liver disease (MASLD) are associated with risk of advanced hepatic fibrosis and cirrhosis. Given the increased risk, the presence of insulin resistance, metabolic syndrome, and hepatic fibrosis should be assessed in individuals with chronic hepatitis B. The Lipoprotein Insulin Resistance Index (LP-IR) is a simple method of assessing insulin resistance in the general population, and lipoprotein profiles have been shown to reflect hepatic steatosis and fibrosis in patients with MASLD. However, LP-IR and lipoprotein profiles have not been evaluated in patients with chronic hepatitis B. **Methods**: Lipoprotein profiles and LP-IR scores were evaluated in a cohort of patients with chronic hepatitis B using NMR spectroscopy. **Results**: Serum samples from 50 patients with chronic hepatitis B and 44 with chronic hepatitis B and metabolic syndrome were analyzed. A cut-off value was defined that differentiates patients with and without insulin resistance. Increased large VLDL and reduced small VLDL particles were observed, suggesting the presence of hepatic steatosis and fibrosis. **Conclusions**: In this largely Asian cohort of chronic hepatitis B patients, where BMI and conventional markers are less reliable, LP-IR scores differentiated patients with and without insulin resistance and metabolic syndrome. In addition, distinctive lipoprotein profiles such as increased large VLDL and reduced small VLDL particles were observed in these chronic hepatitis B patients, suggesting the presence of hepatic steatosis and fibrosis. Future studies with larger sample sizes are necessary to confirm the utility of these NMR-measured lipoprotein parameters in predicting metabolic disease and fibrosis among patients with underlying liver disease.

## 1. Introduction

Both chronic hepatitis B and metabolic dysfunction-associated steatotic liver disease (MASLD) are significant public health problems [1,2,3]. MASLD can present with hepatic steatosis alone or metabolic dysfunction-associated steatohepatitis (MASH) with progression to increased hepatic inflammation and fibrosis [4]. Chronic hepatitis B and MASLD are the most common causes of chronic liver disease worldwide [5]. They are associated with an increased risk of advanced hepatic fibrosis, hepatocellular carcinoma (HCC), and mortality [2,6,7,8]. In North America, about 40% of patients with chronic hepatitis B have MASLD [3,9], and the majority are Asian [6,10,11,12]. Metabolic syndrome and type 2 diabetes (T2D), significantly increase the risk of MASLD [11,12,13]. Khalili and coworkers noted a high prevalence of T2D (12.5%) and impaired fasting glucose (IFG, 7.8%) in a large multiethnic cohort of patients with chronic hepatitis B in North America [13]. As insulin resistance with impaired glucose metabolism is a prominent feature of metabolic syndrome, and advanced liver fibrosis has been associated with metabolic syndrome, a higher risk of hepatic fibrosis progression and carcinogenesis in these individuals should not be underestimated [14,15].

The development of insulin resistance occurs in parallel with changes in the composition and size of lipoprotein particles [13,16]. Insulin resistance leads to an inhibition of lipoprotein lipase (LPL) which, along with increased cholesteryl ester transfer protein (CETP) activity, causes an increase in circulating small low-density lipoprotein (LDL) and large very-low-density lipoprotein (VLDL) particles [17,18]. Inflammation from hepatitis B virus (HBV) infection may further contribute to the impairment of the hepatic insulin signaling pathway, thus promoting insulin resistance and its consequences.

Lipoprotein subclass particle concentrations and mean particle diameters can be determined by proton nuclear magnetic resonance (NMR) spectroscopy [19]. NMR lipoprotein analysis has been used to develop the Lipoprotein Insulin Resistance Index (LP-IR) which takes into account the changes that occur in six lipoprotein parameters, namely, VLDL size, large VLDL particle number (VLDL-P), low density lipoprotein (LDL) size, small LDL-P, high density lipoprotein (HDL) size and large HDL-P [16,20]. The scores generated by LP-IR are positively correlated with insulin resistance as measured by HOMA-IR across different racial groups in the Multi-Ethnic Study of Atherosclerosis (MESA) and verified independently by glucose disposal rates measured by hyperinsulinemic-euglycemic clamps in insulin-sensitive, insulin-resistant, and untreated patients with T2D [20]. LP-IR detects insulin resistance even in lean individuals, thereby identifying insulin resistance independently of body mass index (BMI) and in African Americans who tend to have lower hepatic insulin sensitivity [20]. Hence, LP-IR may also be helpful in the early identification of insulin resistance in ethnicities whose body-fat distribution and anthropometric measurements do not accurately predict metabolic disorders [20,21,22].

It is challenging to identify MASLD and MASH among patients with chronic hepatitis B without liver biopsy results since both entities can cause abnormal serum aminotransferases and increased hepatic fibrosis. MASLD is associated with elevated fasting triglycerides, increased non-HDL, and decreased HDL cholesterol. Recently, several groups have reported associations between MASH and atherogenic lipoprotein profiles [21,23]. Interestingly, Jiang et al. observed a near-linear relationship between mean VLDL particle size and MASLD activity score (MAS) using an analysis of the lipoprotein profiles by NMR. A decrease in small VLDL particle concentration and an increase in large VLDL size were associated with more advanced liver fibrosis compared to liver biopsy [24].

Determining strategies to identify subjects with insulin resistance and the risk of MASLD among patients with chronic hepatitis B, especially in Asians with chronic hepatitis B where demographic characteristics such as BMI are not predictive, is important to enable early intervention and optimal management to prevent liver disease progression. While LP-IR scores are accurate in detecting insulin resistance in the general population, they have not been evaluated in patients with known underlying liver diseases such as chronic hepatitis B. The goals of this study were (1) to evaluate the use of LP-IR scores for assessing insulin resistance in chronic hepatitis B patients, (2) to define a cut-off value for LP-IR that differentiates patients with chronic hepatitis B with and without insulin resistance and metabolic syndrome, the precursor of MASLD, and (3) to evaluate if patients with chronic hepatitis B have modified VLDL subclass concentrations indicative of liver fibrosis.

## 2. Materials and Methods

### 2.1. Subjects

This is a cross-sectional retrospective study where electronic medical records (EMR) were identified during 2011–2019 using ICD-10 coding for chronic hepatitis B at the Beth Israel Deaconess Medical Center (BIDMC). A total of 1543 medical record numbers (MRNs) were identified to have a diagnosis of HBV infection at the Liver Center of BIDMC. Subsequently, we performed individual chart reviews of the hepatitis B patients sequentially to identify those with metabolic syndrome and NAFLD/NASH (accessed before the nomenclature change to MASLD/MASH). This was a non-biased approach for subject identification without preselection of the patients seen at the BIDMC clinic. Among those who met the study criteria, 94 had an adequate amount of fasting serum samples in the BIDMC biorepository. Metabolic syndrome was defined per the National Cholesterol Education Program (NCEP) Adult Treatment Panel III (ATP III) criteria [25]. Three of the following five factors were necessary to establish the diagnosis: Abdominal obesity (≥102 and ≥88 cm for males and females, respectively), elevated triglycerides (≥150 mg/dL) or presence of treatment for this disorder, reduced HDL cholesterol (<40 and <50 mg/dL in males and females, respectively), elevated blood pressure (>130/85 mmHg or using blood pressure medication for hypertension), or elevated fasting glucose (glucose 100–125 mg/dL) [25]. T2D was defined per the American Diabetes Association (ADA) criteria [26]. We considered that a patient had T2D if he/she had been previously diagnosed and acknowledged by the patient’s treating physician in the patient notes, if the patient used diabetic medications, or if fasting blood glucose was ≥126 mg/dL, random blood glucose was ≥200 mg/dL or if HbA1c was ≥6.5%. Patients with other forms of chronic liver diseases or alternative causes for fatty liver disease, such as medication, hepatitis A, C, or D, human immunodeficiency virus (HIV), hereditary hemochromatosis, autoimmune hepatitis, significant alcohol consumption defined as >20 g/day, history of any type of malignancy, and solid organ or bone marrow transplant were excluded from the study. Pregnant patients were also excluded. A total of 94 patients with chronic hepatitis B were selected for this study. The patients with insulin resistance (IR-HBV) groups included a total of 44 patients, 20 with T2D (DM-HBV) and 24 with metabolic syndrome (MS-HBV). The DM-HBV and MS-HBV subgroups are not mutually exclusive. All subjects with T2D will meet the definition of metabolic syndrome. However, none of the subjects that were defined with metabolic syndrome met the definition of having T2D. The control group (C-HBV) included 50 patients. This study has been approved by the BIDMC institutional review board (IRB) protocol #2008P000299 with the most recent approval date of 1 June 2025. This study was conducted in accordance with the Declaration of Helsinki.

### 2.2. Clinical Biochemistry and Measurements of Lipoprotein Profiles

**Figure 1:**
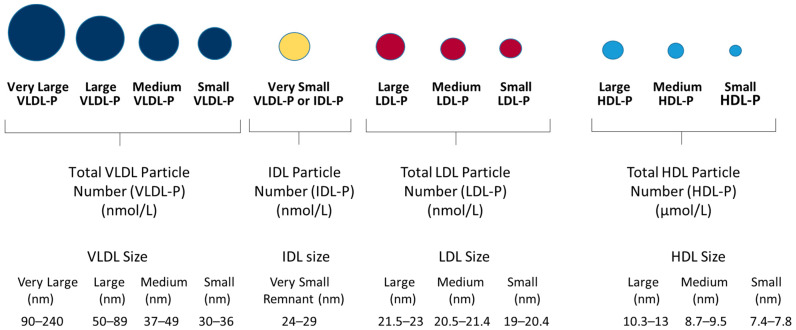
Lipoprotein particle classes (VLDL, LDL, HDL) and subclasses (very large, large, medium, small, very small) reported by the LP4 algorithm along with the range of particle diameters for each of the lipoprotein particle subclasses. VLDL particles are reported as triglyceride-rich lipoprotein (TRL) particles by the LP4 algorithm but are reported here as VLDL to be consistent with previously reported literature.

Routine blood tests, including HbA1c, alanine aminotransferase (ALT), aspartate aminotransferase (AST), hepatitis B surface antigen (HBsAg), hepatitis B e-antigen (HBeAg), and HBV DNA viral loads, were processed using routine testing at the BIDMC clinical laboratory and results were collected from the patient’s EMRs. The results from these tests were collected from the same date that the fasting serum samples were collected, stored, and frozen for future testing purposes. Serum samples for the NMR lipoprotein analysis were thawed, aliquoted (500 μL), and sent to Labcorp (Morrisville, NC, USA) for testing. Labcorp technicians were blinded to the clinical information. Samples were tested on a Vantera^®^ Clinical Analyzer and lipoprotein particle concentrations and sizes were calculated from the measured amplitudes of the spectroscopically distinct signals from each lipoprotein subclass using the LP4 algorithm [27]. Each lipoprotein particle has a unique NMR signature defined by the number of terminal lipid methyl groups, irrespective of core lipid composition, which allows the software algorithm to calculate particle concentrations [19]. Lipoprotein particles, measured using the LP4 algorithm, can be categorized into different classes (VLDL, LDL and HDL) and subclasses (very large, large, medium, small, very small) by their particle diameters (Figure 1). The LP4 algorithm reports the largest lipoprotein particles as triglyceride-rich lipoprotein (TRL) particles. In previous algorithms (e.g., LP3 algorithm), these larger particles were called VLDL. Even though the LP4 algorithm reports “TRL” values, throughout this manuscript they are reported as “VLDL” for consistency with previously reported literature. Mean VLDL, LDL, and HDL particle sizes are weighted averages derived from the sum of the diameters of each subclass multiplied by its relative mass percentage. Insulin resistance was assessed using LP-IR scores, which are calculated based on changes that occur in six lipoprotein parameters: VLDL size, large VLDL particle number (VLDL-P), low density lipoprotein (LDL) size, small LDL-P, high density lipoprotein (HDL) size and large HDL-P [20]. LP-IR scores vary from 0 to 100 (least to most insulin resistant) [20].

### 2.3. Statistical Analyses

The statistical program JMP version 12.0 was used for analyses and reported means ± standard deviation (SD) for the continuous variables. The Shapiro–Wilk test for normality was performed for continuous variables. Categorical variables, such as race and gender, were summarized using frequency and frequency percentage and compared using the Chi-squared (χ^2^) or Fisher’s exact tests. Mann–Whitney–U test was used when continuous variables were not normally distributed. Whenever three subgroups were compared, if the overall Mann–Whitney–U *p*-value was <0.05, the pairwise Steel-Dwass method was performed. SAS version 9.4 was used to conduct univariate logistic regression analyses to ascertain which lipoproteins are associated with insulin resistance. Receiver operating characteristic (ROC) curves were constructed to determine optimal cut-off points for LP-IR. Optimal cut-off points were then selected based on sensitivity, specificity, Youden’s J, and the distance to the point (0, 1) on the ROC curve. To estimate effect sizes, Cohen’s d was used, and 95% confidence intervals (CI) were calculated using the formula provided by Hedges and Olkin (1985) [28]. Associations between continuous variables were assessed using Pearson’s correlation test. For all analyses, *p* values ≤0.05 were considered statistically significant.

## 3. Results

### 3.1. Patient Demographics and Clinical Characteristics

A total of 94 patients with chronic hepatitis B were selected for this study. Patients in the insulin resistance plus chronic hepatitis B (IR-HBV) group included a total of 44 patients: 20 of whom had T2D (DM-HBV) and 24 of whom had metabolic syndrome (MS-HBV). The control group (C-HBV) with chronic hepatitis B only (without insulin resistance or metabolic syndrome) included 50 patients. The total cohort (*n* = 94) had patients of predominantly Asian race (72.3%) and the HBeAg status was negative in 92.6%, indicating a low level of active hepatitis B virus replication and low infectivity at the time of this study. The mean age of the total cohort was 48.4 ± 11.3 years, and 56.4% were male (*n* = 94). There were no significant differences in the patients’ age or race between the IR-HBV and C-HBV groups. The mean BMI was significantly higher in the IR-HBV group than in the C-HBV group (28.1 ± 3.4 vs. 25.0 ± 4.4, *p* < 0.001). Similarly, ALT (38.4 ± 26.2 vs. 27.2 ± 15.6, *p* = 0.01) and AST (27.9 ± 9.4 vs. 24.8 ± 15.6, *p* = 0.07) levels were higher in the IR-HBV group than the C-HBV group. None of the patients had cirrhosis. As expected, the IR-HBV group had higher levels of HbA1c, indicative of lower plasma glucose control (6.2 ± 0.9 vs. 5.3 ± 0.33, *p* < 0.0001). The demographic and clinical characteristics of both groups are summarized in Table 1.

### 3.2. Lipoprotein Analysis by NMR

An analysis was conducted to assess potential differences in the lipoprotein classes and subclasses between chronic hepatitis B patients with (IR-HBV) or without (C-HBV) insulin resistance (Table 2). When compared to the control group (C-HBV), IR-HBV subjects had significantly higher concentrations of total VLDL-P (156.46 ± 59.31 vs. 114.19 ± 60.43, *p* = 0.0009) as well as very large, large, medium, and very small VLDL-P. A closer analysis of the VLDL particles revealed that the concentration of very large VLDL-P was higher in IR-HBV than in C-HBV groups (0.31 vs. 0.14, *p* < 0.001) (Table 2). In contrast, small VLDL-P was lower in IR-HBV compared to C-HBV (42.18 vs. 53.83, *p* = 0.05). While there was no significant difference in the total LDL-P (1132.7 ± 506.5 vs. 1148.9 ± 368.4, *p* = 0.3227) between the two groups, subjects with insulin resistance (IR-HBV) had higher concentrations of small LDL-P and lower concentrations of large and medium LDL-P. IR-HBV subjects had significantly lower concentrations of total HDL-P (15.38 ± 4.99 vs. 17.53 ± 5.59, *p* = 0.0375) which was accompanied by lower concentrations of large and medium HDL-P and no change in small HDL-P. These changes were reflected in the average diameter size of the particles with significantly higher VLDL size and lower LDL and HDL size overall (all *p* < 0.0001) (Table 2). LP-IR scores were also higher in subjects with IR-HBX compared to the control group (C-HBV) (52 ± 14 vs. 31 ± 17, *p* < 0.0001).

**Figure 2:**
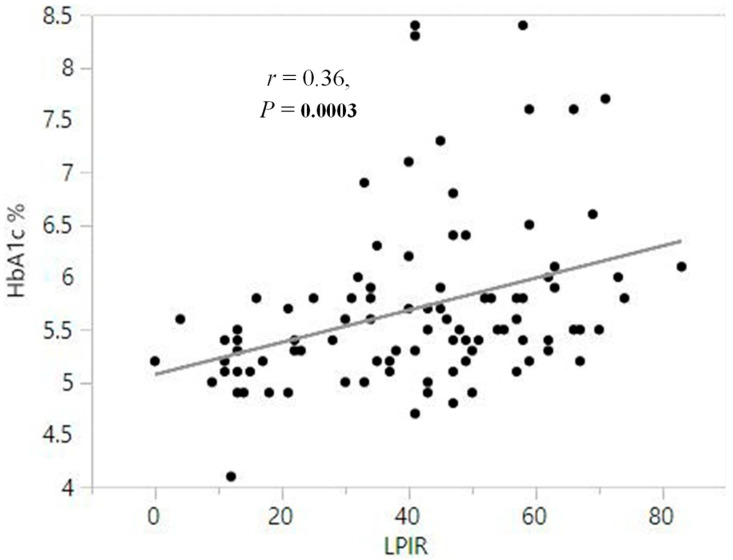
Spearman correlation analysis between HbA1c and Lipoprotein Insulin Resistance Index (LP-IR) scores in the total population of patients with HBV (*n* = 94) illustrating the association between insulin resistance and poor plasma glucose control.

Spearman correlation analysis of the total population of patients with chronic HBV (*n* = 94) revealed a significant positive correlation between LP-IR and HbA1c (r = 0.36, *p* = 0.0003). Figure 2 illustrates the fact that insulin resistance, as assessed by LP-IR scores, is associated with poor plasma glucose control in patients with chronic hepatitis B.

**Figure 3:**
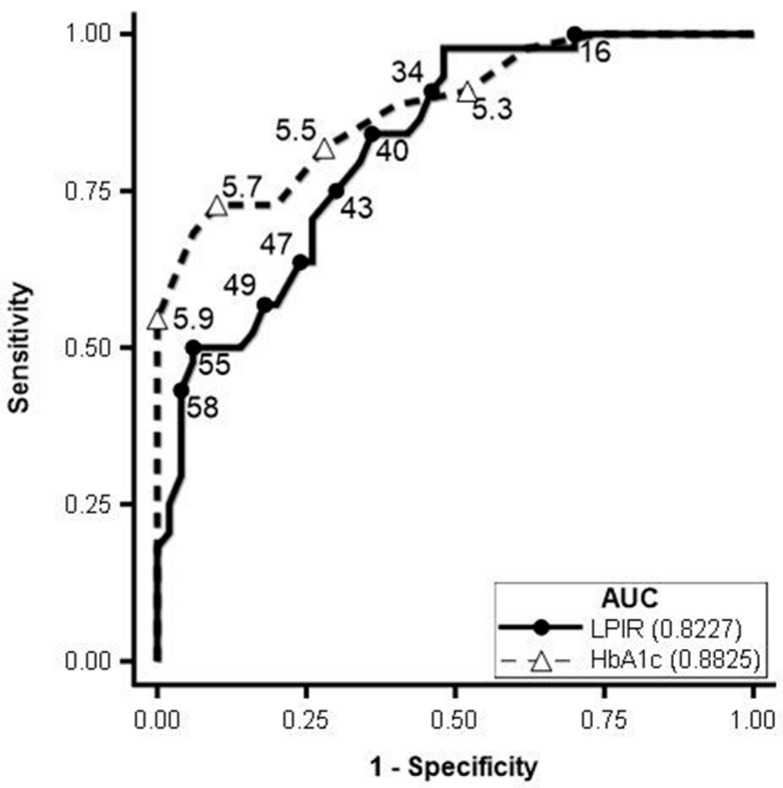
Receiver operating characteristic (ROC) curve for the Lipoprotein Insulin Resistance Index (LP-IR) vs. HbA1c and prediction of metabolic syndrome.

LP-IR scores and the individual components of LP-IR were then used to build receiver operating characteristic (ROC) curves to predict patients with metabolic syndrome. The data revealed that the ROC curves for the six lipoprotein parameters (VLDL size, large VLDL-P, LDL size, small LDL-P, HDL size, and large HDL-P) that comprise LP-IR were not as useful as the composite LP-IR scores. The area under the receiver operating characteristic (AUROC) curve for LP-IR was 0.82 (95% CI: 0.74–0.90). HbA1c had a slightly higher AUROC of 0.88 (95% CI: 0.82–0.95). However, the AUROC for HbA1c was not significantly different from the AUROC for LP-IR (*p* = 0.28). Figure 3 shows a summary of the different cut-off points with Youden’s J along with the ROC curve for LP-IR and HbA1c.

**Figure 4:**
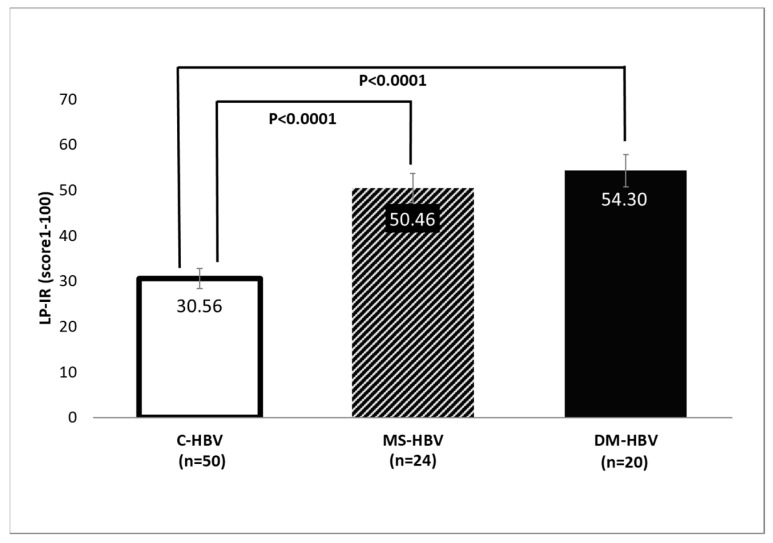
Comparisons of mean LP-IR scores between the C-HBV (*n* = 50), MS-HBV (*n* = 24) and DM-HBV (*n* = 20) groups. Data is reported as mean ± standard error of mean. All variables were compared using Mann–Whitney–U test. If overall *p* values between groups were <0.05, the pairwise Steel-Dwass method was performed.

After evaluating the cut-off points for LP-IR using the highest Youden’s J and the smallest difference between sensitivity and specificity, we found that an LP-IR cut-off point of 43 with a 75% sensitivity and 70% specificity was an optimal marker to differentiate the presence of metabolic syndrome in patients with HBV. To further test this finding, we calculated the LP-IR scores in subjects with chronic hepatitis B only (C-HBV) as well as those with metabolic syndrome (MS-HBV) and those with T2D (DM-HBV). Patients in the C-HBV group had a mean LP-IR value of 31, and all individuals in this group had an LP-IR score below the cut-off of 43. The mean LP-IR score in the MS-HBV and DM-HBV groups were 50 and 54, respectively, both being significantly higher than the mean LP-IR score in the C-HBV group (*p* < 0.0001) (Figure 4).

**Figure 5:**
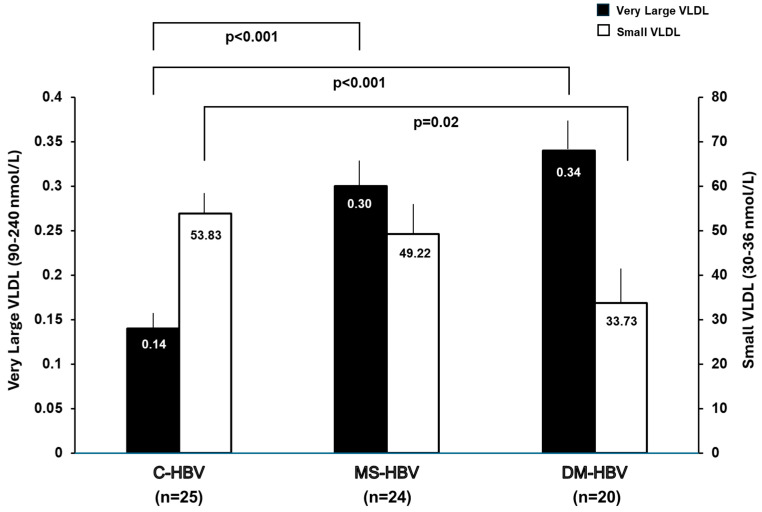
Comparison of the concentrations of very large VLDL and small VLDL particles between the C-HBV (*n* = 50), MS-HBV (*n* = 24), and DM-HBV (*n* = 20) groups. Data is reported as means ± standard error of mean. All variables were compared using Mann–Whitney–U test. If overall P values between groups were <0.05, the pairwise Steel-Dwass method was performed.

Patients in the C-HBV cohort had the lowest levels of very large VLDL-P and the highest levels of small VLDL-P whereas patients in the DM-HBV cohort had the highest levels of very large VLDL-P and lowest levels of small VLDL-P (Figure 5). Although the large and medium HDL-P and LDL-P levels differed between the C-HBV and IR-HBV groups, there were no clear differences in these parameters between the C-HBV, MS-HBV and DM-HBV cohorts (Figure 5).

## 4. Discussion

LP-IR, calculated using NMR technology, is a validated clinical tool to assess insulin resistance in patients with or without metabolic risk factors [20]. We evaluated chronic hepatitis B patients with and without metabolic syndrome or T2D to determine whether LP-IR scores can be applied to patients with underlying HBV infection. To the best of our knowledge, this is the first study to address this question, especially in a largely Asian population where BMI and waist circumference are not good predictors of insulin resistance and metabolic syndrome. The results of this study revealed that LP-IR can detect insulin resistance and metabolic syndrome in a population of largely Asian patients with chronic hepatitis B. This is an important finding because individuals with co-existing chronic hepatitis B and metabolic disease are at higher risk of liver disease progression and mortality [8,29,30,31,32,33]. In addition, the lipoprotein profile results revealed that increased large VLDL and reduced small VLDL particles were observed in patients with chronic hepatitis B, suggesting the presence of hepatic steatosis and fibrosis.

Hepatitis B is one of the major causes of liver-related deaths worldwide. T2D and metabolic syndrome associated with MASLD and MASH can contribute to the progression of liver disease [2,7,10,11,12,15,32,33,34,35]. Once there is inflammation, it is difficult to distinguish whether the liver injury derives from fatty liver disease or HBV infection. Some studies have shown that there may be mechanistic interactions between HBV and the major pathways causing MASLD, which can enhance or interfere with the progression of chronic hepatitis B [8,15,32,33,34,35]. Through the utilization of NMR technology, we were able to differentiate chronic hepatitis B patients with and without metabolic syndrome and T2D; those with metabolic disease having higher LP-IR scores. The results also confirmed that LP-IR was similar to HbA1c, a measure of uncontrolled plasma glucose levels, in predicting metabolic syndrome status in patients with underlying chronic hepatitis B. However, previous studies have also shown that LP-IR scores can assess insulin resistance in patients with fasting plasma glucose levels in the normal range (<100 mg/dL) [20]. Therefore, LP-IR scores would be more clinically useful in patients with HbA1c when HbA1c levels are in the normal range and would be less useful for detecting metabolic syndrome. Moreover, chronic hepatitis B patients with LP-IR levels above the cut-off value of 43 should be further evaluated for coexisting MASLD, MASH and liver fibrosis.

Previous studies focused on MASLD and MASH reported that individuals with T2D were more likely to have significant liver fibrosis and more likely to experience fibrosis progression [15,31,32,36]. Jiang et al. reported a 1.6% decrease in the percentage of small VLDL particles for every stage increase in liver fibrosis in patients with MASLD [24]. We found a similar pattern of decreased small VLDL-P levels in the IR-HBV cohort compared to the C-HBV controls. Furthermore, patients with DM-HBV were at the greatest risk of MASH and advanced liver fibrosis and they had the lowest level of small VLDL-P. Given that mean VLDL size was shown to be associated with MASLD activity in the Jiang et al. study [24], in the current study, higher very large VLDL-P and lower small VLDL-P concentrations suggest that individuals with chronic hepatitis B may also have underlying MASLD activity and liver fibrosis. Ultimately, it is clinically relevant to know whether people with insulin resistance and chronic hepatitis B are at increased risk for liver disease progression and its complications.

This retrospective study has some major limitations that are worth noting, including the cross-sectional nature of the study, which precludes analysis of causation. The number of study subjects was limited since only patients confirmed to have adequate fasting serum volumes in the biorepository were included. The subjects in this study were predominantly Asians. Studies with larger sample sizes from more diverse patient populations should be conducted to confirm the current findings. HBV genotypes were not available for all patients. Since Asians accounted for 72% of the subjects in this study, most subjects in this cohort would likely have HBV genotypes B or C. Hepatic fibrosis data were not uniformly available, which precluded a comprehensive evaluation of the relationship between the lipoprotein parameters and histological findings of the study subjects. Nonetheless, our data provide clinical evidence that LP-IR and NMR lipoprotein analyses are promising, commercially available diagnostic tools that can be used, along with common clinical characteristics, to detect the presence of insulin resistance, metabolic syndrome and liver fibrosis in patients with chronic hepatitis B.

## 5. Conclusions and Future Directions

The results of this study revealed that LP-IR can be used to assess insulin resistance and metabolic syndrome in a population of patients with chronic hepatitis B who were largely of Asian descent where BMI and conventional markers are less reliable. In addition, distinctive NMR lipoprotein profiles such as increased large VLDL and reduced small VLDL particles were observed in patients with chronic hepatitis B, suggesting the presence of hepatic steatosis and fibrosis. Further studies with larger sample size and diverse study populations with and without steatosis and MASH will be required to validate our results and to fully evaluate the diagnostic potential of LP-IR and NMR lipoprotein analysis for hepatic steatosis and fibrosis in patients with underlying HBV infection.

## Figures and Tables

**Table 1:** Demographics and clinical characteristics of the insulin resistance (IR-HBV) and control HBV (C-HBV) cohorts.

	IR-HBV (*n* = 44)	C-HBV (*n* = 50)	Standardized Difference (95% CI)	*p* Value
Age, years	50 ± 13	47 ± 9	0.33 (−0.07–0.74)	0.117
Gender			0.46 (0.05–0.087)	0.03
Male	30 (68.2%)	23 (46.0%)		
Female	14 (31.8%)	27 (54.0%)		
Race			0.21 (−0.20–0.62)	0.32
White	3 (6.8%)	6 (12.0%)		
Black	6 (13.6%)	7 (14.0%)		
Asian	34 (77.3%)	34 (68.0%)		
Hispanic	0 (0.0%)	0 (0.0%)		
Native American/Alaskan	0 (0.0%)	0 (0.0%)		
More than one race	1 (2.3%)	0 (0.0%)		
Unknown/non-reported	0 (0.0%)	3 (6.0%)		
BMI, kg/m^2^	28.1 ± 3.4	25.0 ± 4.4	0.79 (0.37–1.22)	<0.001
ALT, mg/dL	38.4 ± 26.2	27.2 ± 15.6	0.53 (0.12–0.94)	0.01
AST, mg/dL	27.9 ± 9.4	24.8 ± 9.0	0.33 (−0.08–0.74)	0.07
HbA1c, %	6.2 ± 0.9	5.3 ± 0.3	1.45 (0.99–1.90)	<0.0001
HBeAg positive	4 (9.1%)	3 (6.0%)	0.12 (−0.29–0.52)	0.569
On HBV antiviral treatment	21 (47.7%)	21 (42.0%)	0.12 (−0.29–0.52)	0.57

Data are presented as mean ± standard deviation or frequency and frequency percent (%). The Wilcoxon test was used to compare variables that were not normally distributed. Abbreviations: Hepatitis B virus (HBV), confidence interval (CI), body mass index (BMI), alanine aminotransferase (ALT), and aspartate aminotransferase (AST), hepatitis B e-antigen (HBeAg).

**Table 2:** Lipoprotein Analysis by NMR including lipoprotein classes and subclasses in the HBV-Insulin Resistance (IR-HBV) and HBV-Control (C-HBV) groups.

Lipoprotein Classes and Subclasses	IR-HBV (*n* = 44)	C-HBV (*n* = 50)	Standardized Difference (95% CI)	Wilcoxon *p* Value
Total VLDL-P	156.46 ± 59.31	114.19 ± 60.43	0.71 (0.29–1.12)	0.0009
Very Large VLDL-P	0.31 ± 0.20	0.14 ± 0.14	1.03 (0.60–1.47)	<0.0001
Large VLDL-P	2.48 ± 1.61	1.59 ± 1.46	0.58 (0.17–0.99)	0.0045
Medium VLDL-P	14.17 ± 7.93	10.26 ± 5.92	0.56 (0.15–0.98)	0.0153
Small VLDL-P	42.18 ± 33.74	53.83 ± 36.92	−0.33 (−0.74–0.08)	0.0543
Very Small VLDL-P	97.31 ± 60.18	48.38 ± 48.64	0.90 (0.48–1.33)	<0.0001
Total LDL-P	1132.7 ± 506.5	1148.9 ± 368.4	−0.04 (−0.44–0.37)	0.3227
Large LDL-P	315.0 ± 252.4	512.6 ± 253.5	−0.78 (−1.2–−0.36)	0.0003
Medium LDL-P	157.2 ± 288.5	325.6 ± 373.3	−0.50 (−0.91–−0.09)	0.0060
Small LDL-P	660.5 ± 332.0	310.9 ± 244.6	1.21 (0.77–1.65)	<0.0001
Total HDL-P	15.38 ± 4.99	17.53 ± 5.59	−0.40 (−0.81–0.01)	0.0375
Large HDL-P	0.92 ± 0.59	1.93 ± 1.64	−0.80 (−1.22–−0.38)	0.0002
Medium HDL-P	2.29 ± 1.92	3.60 ± 2.07	−0.65 (−1.07–−0.24)	0.0008
Small HDL-P	12.17 ± 3.42	12.00 ± 3.18	0.05 (−0.36–0.45)	0.8157
VLDL Size	47.54 ± 6.35	42.49 ± 4.88	0.90 (0.48–1.33)	<0.0001
LDL Size	20.72 ± 0.78	21.42 ± 0.62	−0.99 (−1.42–−0.56)	<0.0001
HDL Size	8.82 ± 0.30	9.22 ± 0.41	−1.09 (−1.52–−0.65)	<0.0001
LP-IR	52 ± 14	31 ± 17	1.38 (0.93–1.83)	<0.0001

Abbreviations: Hepatitis B virus with insulin resistance (IR-HBV), Hepatitis B virus only control (C-HBV), confidence interval (CI), very-low-density lipoprotein particle number (VLDL-P), low density lipoprotein particle number (LDL-P), high density lipoprotein particle number (HDL-P), Lipoprotein Insulin Resistance Index (LP-IR).

## Data Availability

The original contributions presented in this study are included in the article. Further inquiries can be directed to the corresponding author.

## Abbreviations

The following abbreviations are used in this manuscript:

ALT	Alanine aminotransferase
AST	Aspartate aminotransferase
AUROC	Area under the receiver operating characteristic curve
BIDMC	Beth Israel Deaconess Medical Center
BMI	Body mass index
C-HBV	Patients with hepatitis B virus only
DM-HBV	Patients with hepatitis B virus and type 2 diabetes mellitus
EMR	Electronic medical records
HDL	High density lipoprotein
HOMA-IR	Homeostatic Model Assessment for Insulin Resistance
IR-HBV	Patients with hepatitis B virus and insulin resistance
LDL	Low density lipoprotein
LP-IR	Lipoprotein Insulin Resistance Index
MASH	Metabolic dysfunction-associated steatohepatitis
MASLD	Metabolic dysfunction-associated steatotic liver disease
MRN	Medical record number
MS-HBV	Patients with hepatitis B virus and metabolic syndrome
NMR	Nuclear magnetic resonance spectroscopy
ROC	Receiver operating characteristic curve
T2D	Type 2 diabetes mellitus
VLDL	Very low density lipoprotein
